# Selenium-analogs based on natural sources as cancer-associated carbonic anhydrase isoforms IX and XII inhibitors

**DOI:** 10.1080/14756366.2023.2191165

**Published:** 2023-03-20

**Authors:** Nora Astrain-Redin, Niccolò Paoletti, Daniel Plano, Alessandro Bonardi, Paola Gratteri, Andrea Angeli, Carmen Sanmartin, Claudiu T. Supuran

**Affiliations:** a Department of Pharmaceutical Technology and Chemistry, University of Navarra, Pamplona, Spain; b Department NEUROFARBA – Pharmaceutical and nutraceutical section, Laboratory of Molecular Modeling Cheminformatics & QSAR, University of Firenze, Sesto Fiorentino, Florence, Italy; c Department NEUROFARBA – Pharmaceutical and nutraceutical section, University of Firenze, Sesto Fiorentino, Florence, Italy

**Keywords:** Selenium, carbonic anhydrase, inhibitors, NSAIDs, tumours

## Abstract

In the relentless search for new cancer treatments, organoselenium compounds, and carbonic anhydrase (CA) inhibitors have emerged as promising drug candidates. CA isoforms IX and XII are overexpressed in many types of cancer, and their inhibition is associated with potent antitumor/antimetastatic effects. Selenium-containing compounds, particularly selenols, have been shown to inhibit tumour-associated CA isoforms in the nanomolar range since the properties of the selenium atom favour binding to the active site of the enzyme. In this work, two series of selenoesters (1a–19a and 1b–19b), which gathered NSAIDs, carbo/heterocycles, and fragments from natural products, were evaluated against hCA I, II, IX, and XII. Indomethacin (17b) and flufenamic acid (19b) analogs exhibited selectivity for tumour-associated isoform IX in the low micromolar range. In summary, selenoesters that combine NSAIDs with fragments derived from natural sources have been developed as promising nonclassical inhibitors of the tumour-associated CA isoforms.

## Introduction

Cancer is a major health burden that is increasing every day despite efforts in diagnosis and treatment. In 2020, there were 19.3 million new cancer cases (10.1 million males vs. 9.2 million females), plus 9.9 million cancer deaths (5.5 million males vs. 4.4 million females) worldwide.^1^ The research of new therapeutic targets and the development of novel treatments continues unabated. The emergence of resistance to current drugs further highlights the need to bring new effective therapeutic options to the market. In this context, carbonic anhydrase (CA) inhibitors have emerged as promising anticancer drug candidates.

The CAs are a superfamily of metalloenzymes that reversibly catalyse the conversion of CO_2_ into carbonic acid. There are at least eight families of CA (α-ι classes) and the CA-α are present in humans. The CA enzymes found in mammals fall into four broad subgroups according to their cellular location: cytosolic isoforms (CA-I, CA-II, CA-III, CA-VII, and CA-XIII), mitochondrial isoforms (CA-VA and CA-VB), membrane-bound isoforms (CA-IV, CA-IX, CA-XII, CA-XIV, and CA-XV) and secreted isoforms (CA-VI).^2^ CA inhibitors are clinically used mainly as diuretics and antiepileptics, but novel applications in cancer treatment have been developed. Interestingly, isoforms IX and XII are overexpressed in certain types of cancers and are associated with the growth, migration, invasion, and metastasis of tumors.^3–5^ Therefore, they have been widely studied as new therapeutic targets for cancer.^6–8^ CA-IX is expressed in a restricted number of normal tissues, whereas is highly expressed in many solid tumours, particularly in renal carcinoma.^9^^,^^10^ Moreover, CA-IX is a hypoxia-associated isoform that plays a crucial role in extracellular acidification of the solid tumour, contributing to tumour progression.^11^^,^^12^ On the other hand, CA-XII is overexpressed in a range of human tumours including gastric, ovarian, lung, and brain, whereas its expression in normal tissues is also restricted.^13–15^ In addition, CA-XII expression is induced by oestrogen; hence this isoform is highly expressed in oestrogen receptor-positive breast cancer.^16^ Therefore, it seems promising to develop inhibitors of these two CA tumour-associated isoforms for the treatment of several types of cancer, with one such compound, SLC-0111 in Phase Ib/II clinical trials as an antitumor agent.^17^

Selenium (Se) compounds have been extensively studied as pharmaceutical drugs since they have shown antioxidant,^18^^,^^19^ antitumor,^18^^,^^20^ antiviral,^21^^,^^22^ and antimicrobial^23^ actions. Particularly, selenols have arisen as a powerful scaffold to develop nonclassical inhibitors of the tumour-associated CA isoforms, due to the soft character of the Se atom, the polarity of the Se-H bond, and the affinity of Se-containing compounds for zinc finger proteins.^24^ Aryl selenol derivatives have demonstrated to inhibit CA-IX at nanomolar concentrations, whereas the cytosolic CA isoforms proved to be less sensitive. Moreover, it was shown that selenol was bound directly to the Zn (II) ion from the CA active site with its usual tetrahedral geometry.^25^ Selenol group (R-SeH) is quite nucleophilic and possess a low p*K_a_* value at physiological pH (i.e. 5.4), the main species reported is the anionic selenoate (RSe¯), which can readily react with the zinc ion of the CA active site. In addition, aliphatic selenols were synthesised and evaluated as CA inhibitors, and they demonstrated to be more potent against the cytosolic CAs rather than the membrane and tumour-associated isoforms.^26^ However, the main limitation of selenols is that they are very unstable, showing a high tendency to be oxidised to diselenides. An approach to solve this problem is the development of selenoesters, which by the action of an esterase enzyme will release the selenol group and the corresponding carboxylic acid. The Supuran’s group has demonstrated the thioesterase,^27^ esterase,^28^ and selenoesterase^29^ activities of human CA isoforms. Therefore, it seems reasonable to design selenoesters with the aim of creating a prodrug that, after the action of CA (with selenoesterase function), releases the active selenol. This strategy also makes it possible to introduce into the molecule another active fragment, in addition to selenol, which will be released in the form of carboxylic acid.

**Figure 1.**
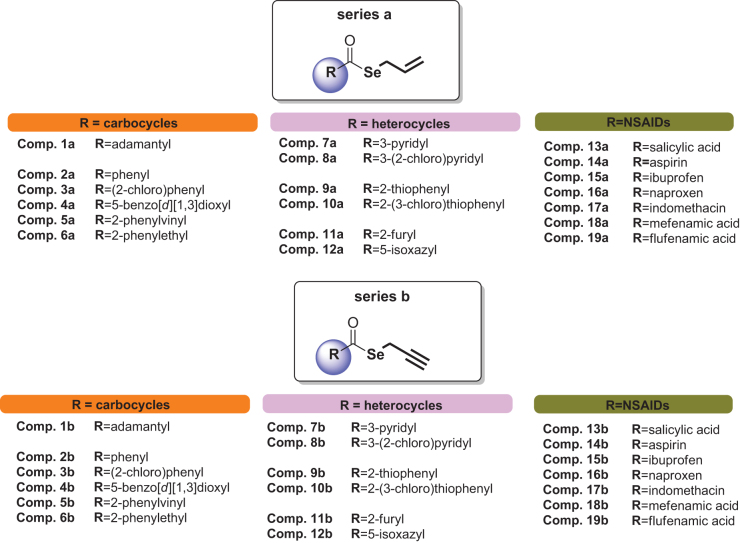
Chemical structures of synthetic compounds **1a**–**19a** and **1b**–**19b**.^30^

**Figure 2.**
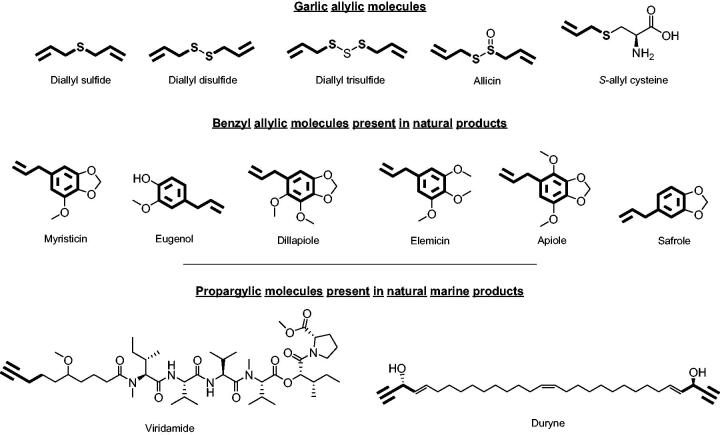
Allylic and propargylic molecules of natural products.^30^

Herein, we report 38 selenoesteres (Figure 1), combining active fragments from natural sources with non-steroidal anti-inflammatory drugs (NSAIDs) and carbo- and hetero-cycles derivatives as inhibitors of tumour-associated human CA. The part of the molecule corresponding to the selenol, that might be released, is an allylic (series **a**) or propargylic (series **b**) chain. This side of the molecule has been designed inspired by the active fragments present in natural sources (Figure 2).

The allylic chain is present in active garlic derivatives such as diallyl selenides, allicin or *S*-allyl cysteine. It is also found in benzyl allylic derivatives present in several plants such as cloves, curcuma, anise, or parsley.^31^ On the other hand, the active propargylic fragment can be found in natural marine products such as viridamide (isolated from cyanobacteria) and duryne (metabolite produced by one type of marine sponge). The other part of the molecule, which ought to be released in the form of carboxylic acid, are carbo- and hetero-cycles derivatives and NSAIDs. Inflammation is recognised as a hallmark feature of cancer development and progression,^32^ thus the incorporation of NSAIDs into the molecule provides an extra activity that may enhance their profile as tumour-associated CA isoforms inhibitors. Moreover, NSAIDs are carboxylic acid-based compounds that have been reported as a new family of nonclassical CA inhibitors.^33–35^ Likewise, carbo- and hetero-cycles derivatives, such as quinazoline- or benzodioxol-based compounds, have been demonstrated to inhibit some isoforms of CA, including tumour-associated IX and XII.^36^^,^^37^

## Experimental

### Chemistry

The studied compounds **1a–19a** and **1b–19b** were synthesised in our previous work^30^ and their chemical structures are depicted in Figure 1.

### Carbonic anhydrase inhibition studies

An Applied Photophysics stopped-flow instrument has been used for assaying the CA catalysed CO_2_ hydration activity.^38^ Phenol red (at a concentration of 0.2 mM) has been used as an indicator, working at the absorbance maximum of 557 nm, with 20 mM Hepes (pH 7.5) as a buffer and 20 mM Na_2_SO_4_ (for maintaining constant the ionic strength), following the initial rates of the CA-catalysed CO_2_ hydration reaction for a period of 10–100 s. The CO_2_ concentrations ranged from 1.7 to 17 mM for the determination of the kinetic parameters and inhibition constants. For each inhibitor, at least six traces of the initial 5 − 10% of the reaction have been used for determining the initial velocity. The uncatalyzed rates were determined in the same manner and subtracted from the total observed rates. Stock solutions of inhibitor (0.1 mM) were prepared in distilled − deionized water and dilutions up to 0.01 nM were done thereafter with the assay buffer. Inhibitor and enzyme solutions were preincubated together for 15 min at room temperature before assay to allow for the formation of the E − I complex. The inhibition constants were obtained by nonlinear least-squares methods using PRISM 3 and the Cheng − Prusoff equation, as reported earlier,^39^^,^^40^ and represent the mean from at least three different determinations. The enzyme concentrations were in the range 3–11 nM. All human CA (hCA) isoforms were recombinant ones obtained in-house as reported earlier.^41^^,^^42^

### Molecular modelling

The crystal structures of CA I (PDB:2NMX),^43^ CA II (PDB:3K34),^44^ CA IX (PDB:5FL4),^45^ and CA XII (PDB:1JD0)^46^ used for computational studies were downloaded by Protein Data Bank^47^ and prepared according to the Protein Preparation module in Maestro Schrödinger suite, assigning bond orders, adding hydrogens, deleting water molecules, and optimising H-bonding networks. Finally, energy minimisation with a Root Means Square Deviation (RMSD) value of 0.30 was applied using an Optimised Potential for Liquid Simulation (OPLS4) force field.^25^^,^^48–51^ Grids for docking were centred in the centroid of the complexed ligand. Docking studies were carried out with the program Glide^48^(f) using the standard precision (SP) mode. 3D ligand structures were prepared by Maestro^48^(a). QM geometry optimisation and atomic electrostatic charges computation were performed with Jaguar^48^(g) fitting them to an electrostatic potential calculated at the B3LYP/cc/pvtz-f level of theory. ESP atomic charges were used in docking simulations. The OPLS4 force field was modified according to Schrödinger to enable negatively charged selenate moieties for the docking procedure in Glide. Indeed, the OPLS FF does not support parameters for the Lewis structure of selenates. The best docking pose for each compound was submitted to DFT (B3LYP/LACVP*+) calculations with Jaguar within both built model systems. The QM-optimised ligands were rescored in the complete hCA macromolecular environment with Glide^48^(f).

Molecular dynamics (MD) simulations were performed using Desmond Molecular Dynamics System (v.6.7)^48^€ (Schrödinger suite) and OPLS4 force field. All systems were solvated in an orthorhombic box using simple point charge water molecules extended 15 Å away from any protein atom. The system was neutralised with 0.15 M Cl^−^ and Na^+^ ions. The simulation protocol included a starting relaxation step followed by a final production phase of 100 ns. In particular, the relaxation step comprised the following: (a) a stage of 100 ps at 10 K retaining the harmonic restraints on the solute heavy atoms (force constant of 50 Kcal/mol/Å^2^) using the NPT ensemble with Brownian dynamics; (b) a stage of 12 ps at 10 K with harmonic restraints on the solute heavy atoms (force constant of 50 Kcal/mol/Å^2^), using the NVT ensemble and Berendsen thermostat; (c) a stage of 12 ps at 10 K and 1 atm, retaining the harmonic restraints and using the NPT ensemble and Berendsen thermostat and barostat; (d) a stage of 12 ps at 300 K and 1 atm, retaining the harmonic restraints and using the NPT ensemble and Berendsen thermostat and barostat; (e) a final 24 ps stage at 300 K and 1 atm without harmonic restraints, using the NPT Berendsen thermostat and barostat. The final production phase of MD was run using a canonical NPT Berendsen ensemble at 300 K. During the MD simulation, a time step of 2 fs was used while constraining the bond lengths of H atoms with the M-SHAKE algorithm. The atomic coordinates of the system were saved every 100 ps along the MD trajectory. Protein RMSD, ligand RMSD/RMSF (Root Mean Square Fluctuation) ligand torsions evolution, and occurrence of intermolecular H-bonds and hydrophobic contacts were provided by the Simulation Interaction Diagram (SID) implemented in Maestro along with the production phase of the MD simulation. The tool reads the MD trajectory file and identifies ligand/target interactions repeatedly occurring during the simulation time (for instance, a 60% value suggests that the interaction is maintained for 60% of the MD). The 1000 frames resulting from MDs were clustering using the Conformer Cluster tool implemented in the Schrödinger suite in 10 clusters. The representative poses of the most abundant clusters were refined with Prime MM-GBSA calculations (v.5.5)^48^(d) with a VSGB (Variable Surface Generalised Born) solvation model considering the target flexible within 3 Å around the ligand. Figures were generated with Maestro and Chimera.^48^^,^^52^

## Results and discussion

### Chemistry

**Scheme 1.**
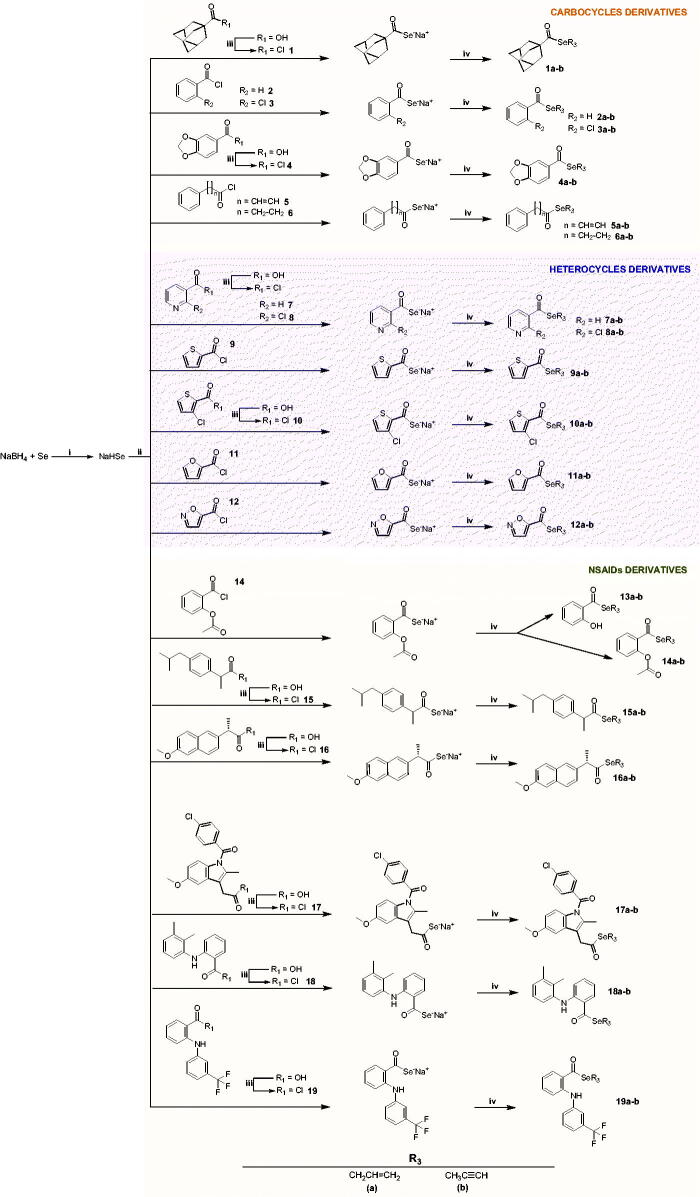
Synthesis procedure to yield **1a–19a** and **1b–19b** derivatives. Reagents and conditions: (i) H_2_O, 20 min, and room temperature; (ii) THF/H_2_O, 60 min, and room temperature; (iii) ClCOCOCl, CH_2_Cl_2_, 12 h, and room temperature; (iv) BrCH_2_CH = CH_2_ or BrCH_2_C≡CH, THF/H_2_O, 90 min, and room temperature.^30^

The synthesis method of the compounds is shown in Scheme 1. The synthesis procedure and the characterisation of the compounds were described in our previous study.^30^ Briefly, hydrogen sodium selenide (NaHSe) was formed by the reduction of elemental Se with NaBH_4_ in water. Then, the corresponding acid chloride was added to the reaction mixture to form the corresponding sodium selenoate by a nucleophilic acyl substitution. Finally, the target compounds were obtained through a nucleophilic substitution over the allyl/propargyl bromide. All the compounds were purified by column chromatography.

### Carbonic anhydrase inhibition

All the compounds **1a–19a** and **1b–19b** were tested *in vitro* for the inhibitory activity against hCA isoforms I, II, VA, IX, and XII, and their activities were compared to the standard CA inhibitor acetazolamide (AZZ) (Table 1). Among the compounds, 9 of 38 inhibited at least one CA isoforms with Ki values in the low micromolar range and the following structure-activity relationship (SAR) may be observed regarding inhibition data in Table 1:

**Table 1.** Inhibition of hCA I, II, VA, IX, and XII activity for compounds **1a–19a** and **1b–19b**.

	*K*_i_ (μM)					*K*_i_ (μM)			
Compound	hCA I	hCA II	hCA IX	hCA VA	Compound	hCA I	hCA II	hCA IX	hCA XII
**1a**	>100	>100	>100	>100	**1b**	>100	>100	9.1	28.4
**2a**	>100	>100	>100	92.3	**2b**	80.5	>100	7.8	55.7
**3a**	>100	>100	>100	94.2	**3b**	42.1	>100	41.6	28.8
**4a**	n.d	n.d	n.d	n.d	**4b**	31.3	53.5	67.7	>100
**5a**	>100	>100	>100	86.3	**5b**	24.1	42.7	79.3	>100
**6a**	n.d	n.d	n.d	n.d	**6b**	3.1	9.5	25.0	7.0
**7a**	>100	>100	>100	>100	**7b**	30.2	66.3	38.2	85.1
**8a**	>100	>100	89.4	96.5	**8b**	2.2	8.6	48.5	9.6
**9a**	>100	>100	>100	>100	**9b**	9.2	46.6	>100	97.0
**10a**	>100	>100	>100	96.7	**10b**	>100	88.5	>100	98.5
**11a**	>100	>100	>100	>100	**11b**	8.5	49.9	7.1	59.6
**12a**	>100	>100	>100	>100	**12b**	58.4	>100	22.4	66.6
**13a**	>100	>100	>100	>100	**13b**	23.3	>100	54.8	>100
**14a**	>100	>100	>100	>100	**14b**	5.0	55.3	>100	89.7
**15a**	n.d	n.d	n.d	n.d	**15b**	77.1	>100	47.7	>100
**16a**	>100	>100	>100	>100	**16b**	91.5	>100	24.7	>100
**17a**	n.d	n.d	n.d	n.d	**17b**	>100	>100	58.5	>100
**18a**	n.d	n.d	n.d	n.d	**18b**	>100	4.5	9.6	46.3
**19a**	n.d	n.d	n.d	n.d	**19b**	>100	>100	9.5	>100
**AZZ**	0.25	0.012	0.026	n.d	**AZZ**	0.25	0.012	0.026	n.d

nd: No data

- Surprisingly, the allylic chain (series **a**) presence in all the compounds leads to a loss of activity in the tested CA isoforms. On the contrary, the introduction of the propargylic chain (series **b**) seems important for the compounds’ inhibitory capacity since *K*_i_ values lower than 10 µM have been observed in some cases.
- Among the compounds of series **b**, the following SAR may be noted:
  1. The cytosolic CA I was inhibited by the aromatic derivative **6b** which has a two-carbon atom spacer between the benzene ring and the carbonyl group (*K*_i_ = 3.1 μM). This compound was 7.7 and 26 times more potent than its analog with the doble-bond spacer (compound **5b**: *K*_i_ = 24.1 μM) and with the analog without the spacer (compound **2b**: *K*_i_ = 80.5 μM), respectively. In addition, the presence of a chlorine atom in the *ortho* position (**3b**) or the presence of an oxygen atom in both 3 and 4 positions (**4b**) improved the inhibition capacity of the benzene-derived compounds (compound **3b** and **4b**: *K*_i_ = 42.1 and 31.3 μM, respectively). However, the substitution of the benzene ring by a carbocycle, such as an adamantane, led to the loss of activity (compound **1b**: *K*_i_ >100 μM). On the other hand, the inhibition potency increased with the presence of heterocycles such as pyridine (**7b**), thiophene (**9b**), and furan (**11b**) (*K*_i_ = 8.5–30.2 μM). It is curious how the pyridine derivative with one chlorine atom in the *ortho* position increased the inhibitory activity 13.7 times (compound **8b:** *K*_i_ = 2.2 μM) compared to unsubstituted pyridine, while the *ortho*-substituted thiophene derivative with one chlorine atom lost the activity (compound **10b**: *K*_i_ >100 μM). The substitution of the furan ring (**11b**) by the isoxazole one (**12b**) decreased the inhibitory capacity nearly 7-fold (*K*_i_ = 8.5 and 58.4 μM, respectively). Finally, among the NSAID derivatives, the aspirin analog (**14b**) was the most active, with a *K*_i_ value of 5.0 μM, followed by the salicylic derivative (**13b**), *K*_i_ = 23.3 μM. The remaining analogs showed no inhibition capacity (*K*_i_ > 77 μM).
  2. The dominant cytosolic human isoform, CA II, was less sensitive to the selenoester derivatives. As in CA isoform I, the aromatic derivative **6b**, which has a two-carbon atom spacer between the benzene ring and the carbonyl group, and the compound **8b**, with a chlorine atom substituted pyridine derivative, inhibited CA II in the low micromolar range (*K*_i_ = 9.5 and 8.6 μM, respectively). The adamantane derivative (**1b**), as well as the benzene analogue (**2b**), did not inhibit this isoform. The benzene derivatives substituted with the chlorine atom (**3b**) and with the oxygen atoms (**4b**) were less active in this isoform, as was the derivative with the double bond spacer (**5b**). Derivatives containing unsubstituted heterocycles inhibited CA II in the medium micromolar range (compounds **7b**, **9b**, and **11b**: *K*_i_ = 66.3, 46.6, and 49.9 μM, respectively). The presence of the isoxazole ring (**12b**) led to the loss of inhibition activity (*K*_i_ > 100 μM). Likewise, the aspirin derivative (**14b**) inhibited CA II 11 times less than against CA I and the salicylic analog (**13b**) was not able to inhibit this CA isoform. The rest of the NSAID derivatives did not inhibit CA II, except for the mefenamic acid analog (**18b**), which exhibited 22-fold greater inhibition potency than for CA I isoforms (*K*_i_ = 4.5 μM).
  3. The tumour-associated transmembrane CA IX was inhibited by compounds **1b** in the low micromolar range (*K*_i_ = 9.1 μM). This adamantane derivative was not able to inhibit cytosolic isoforms I and II. Similarly, the benzene analog (**2b**), which had not inhibited isoforms I and II, exhibited a potent inhibition action with a *K*_i_ value of 7.8 μM (nearly 13 and 10 times higher than for isoforms I and II, respectively). The remaining benzene derivatives (**3b** and **4b–6b**) inhibited CA IX in the medium-high micromolar range (*K*_i_ = 25.0–79.3 μM). Furthermore, the unsubstituted thiophene derivative (**9b**), which inhibited CA I in the low micromolar range, did not inhibit CA IX. In addition, the presence of the thiophene ring (**9b**) did not provide inhibition activity, in contrast to what happened in isoform I (*K*_i_ = 9.2 μM), while the presence of the furan ring (**11b**) maintained the inhibition potency in the low micromolar range (*K*_i_ = 7.1 μM). On the other hand, the NSAID derivatives showed greater activity against this isoform (*K*_i_ = 9.5–58.5 μM), except for the aspirin analog (**14b**) which exhibited no inhibition capacity. The phenamate derivatives (**18b** and **19b**) stood out with *K*_i_ values of 9.6 and 9.5 μM, respectively.
  4. As in CA isoforms I and II, both aromatic derivative **6b**, which has a two-carbon atom spacer between the benzene ring and the carbonyl group, and compound **8b**, with a chlorine atom substituted pyridine derivative, inhibited tumour-associated CA XII in the low micromolar range (*K*_i_ = 7.0 and 9.6 μM respectively). It is very remarkable the fact that the introduction of a double bond in the two-carbon spacer (**5b**) led to the loss of CA XII inhibition activity. The introduction of the adamantane carbocycle (**1b**) or the unsubstituted or chloro-substituted benzene ring (**2b** and **3b** respectively) maintained the inhibition potency in the medium micromolar range (*K*_i_ = 28.4–55.7 μM). However, the replacement of the benzene ring by pyridine (**7b**) or benzodioxol (**4b**) resulted in the loss of inhibition activity. In addition, thiophene derivatives (**9b** and **10b**) did not inhibit CA IX and furan and isoxazole derivatives (**11b** and **12b**, respectively) did so in the medium-high micromolar range (*K*_i_ = 59.6 and 66.6 μM respectively). No NSAID derivative was able to inhibit CA IX, except for the **18b** compound, the mefenamic acid analog, which did so in the medium micromolar range (*K*_i_ = 46.3 μM).
- An interesting inhibition profile was observed for indomethacin and flufenamic acid analogs (**17b**) and (**19b**), respectively, which exhibited selectivity for tumour-associated isoform IX. Likewise, adamantane analog (**1b**) showed selectivity for tumour-associated isoforms IX and XII. On the other hand, salicylic (**13b**), naproxen (**16b**), and ibuprofen (**15b**) analogs exhibited selectivity for cytosolic CA I isoform and tumour-associated CA IX. Another interesting point was that the unsubstituted **(7b**) and chlorine substituted (**8b**) pyridine and furane (**11b**) heterocyclic derivatives, as well as the benzene derivative with the two-carbon spacer (**6b**), showed inhibition activity in all CA isoforms. Compounds **8b** and **6b** inhibited all CA isoforms in the low-medium micromolar range (*K*_i_ = 2.2–48.5 μM).

### Molecular modelling

*In silico* studies were applied to get insight into the different inhibitory activities of derivatives in Table 1 towards the CA I and II (off-target) and the tumour-associated isoforms CA IX and CA XII. As already reported, the investigated compounds act as prodrugs that, thanks to the esterase activity of CAs, release selenol and carboxylic fragments. Thus, interaction studies were carried out on the active allyl (**a**) and propargyl (**b**) selenol moieties of compounds **1–19** using the available 3D coordinates of the targets.

**Figure 3.**
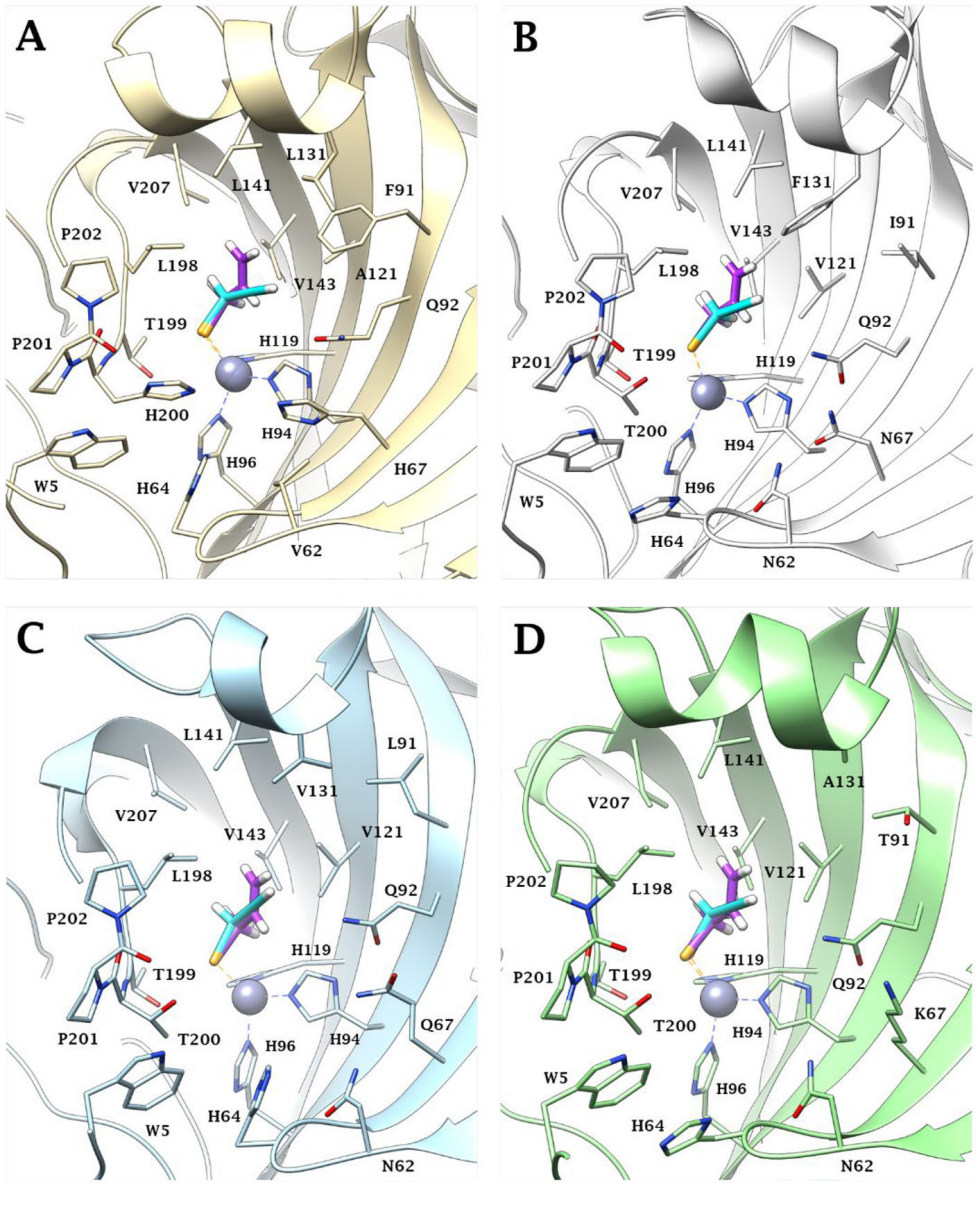
Predicted binding mode of propargyl selenol (violet) and allyl selenol (cyan) within the (A) CA I (yellow), (B) CA II (white), (C) CA IX (blue) and (D) CA XII (green) active site.

According to the literature,^25^^,^^53^ all docking solutions have shown the deprotonated Se atom bound to the zinc atom in tetragonal coordination in the active site of the investigated CA isoforms (Figure 3).

Both **a** and **b** selenol actives are positioned in an area featured by conserved amino acid residues in the different CA isoforms, positioning in the same manner in all the studied isoenzymes. In detail, the allyl selenol obtained from the hydrolysis of selenoesters of series **a**, orients in a cleft, lined by hydrophobic residues (A121 (CA I)/V121 (CA II, IX, XII); L131 (CA I)/F131 (CA II)/V131 (CA IX)/A131 (CA XII); L141, V143, L198 and V207), while the propargyl end points towards the hydrophilic half of the CAs active sites, defined by residues Q92, H67 (CA I)/N67 (CA II)/Q67 (CA IX)/K67 (CA XII), V62 (CA I)/N62 (CA II, IX, XII), H64 and W5.

**Figure 4.**
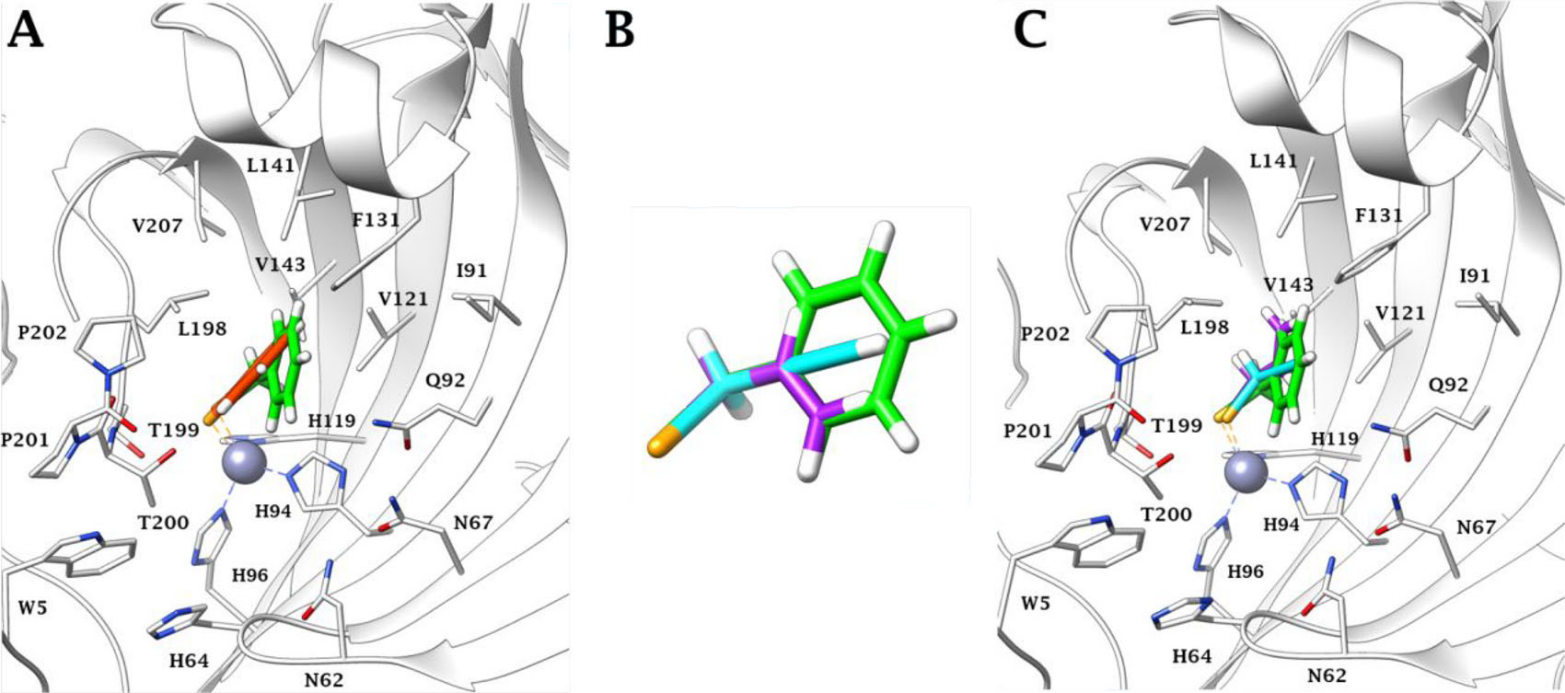
(A) Superposition of (A) X-ray solved structures of phenyl selenol-CA II (red, PDB:6HX5)^25^ and benzyl selenol-CA II (green, PDB:7QBH)^53^ complex. (B) Superposition of ligands benzyl selenol (green), propargyl selenol (cyan), and allyl selenol (violet). (C) Superposition of X-ray solved structures of benzyl selenol-CA II (green)^53^ and predicted binding mode of allyl (violet)/propargyl (cyan) selenol within CA II active site.

As is evident by Figure 4, the geometric parameters (bond length and angles) of the experimental (X-ray data) and the *in silico* (docking poses) solutions of the **a** (allyl) and **b** (propargyl) actives, are in very good agreement (Table 2). 100 ns long molecular dynamics simulations confirm the stability over the time of the docking solutions.

**Table 2.** Geometric parameters of docking and crystallography data.

	Se-Zn bond length (Å)	Zn-Se-C angle (°)
a-selenol (allyl)	2.54	102.7
b-selenol (propargyl)	2.53	105.2
6HX5 (phenyl selenol)	2.51	108.9
7QBH (benzyl selenol)	2.50	99.0

The wider network of interactions of the **b** compared to **a** active does not, as such, explain the large different activity pointed out by inhibition studies, thereby supporting the previous hypothesis that the difference in the activity between derivatives of the two studied series is due to a different hydrolysis rate of the compounds in the CA isozymes. The more compact conformation assumed by derivatives **1–19b** could allow an easier entry into the active sites, promoting faster hydrolysis and leading to a higher inhibitory activity compared to analogs of series **a**.

## Conclusions

Novel 38 Se-analogs which present an innovative structural design have been tested as CA inhibitors. Their design takes advantage of the selenoesterase activity that CA has demonstrated,^29^ as they combine two active fragments via a selenoester functional group which will be release after the action of CA. In this work, propargyl derivatives have exhibited greater *in vitro* inhibitory activities against hCA I, hCA II, hCA XI, and hCA XII, rather than the allyl derivatives. Indomethacin (**17b**) and flufenamic acid (**19b**) analogs exhibited selectivity for tumour-associated isoform IX in the low micromolar range. Likewise, adamantane analog (**1b**) showed selectivity for tumour-associated isoforms IX and XII. These results seem to suggest that molecules containing carbocycles are more selective than the ones which present heterocycles, providing a new approach for the investigation of future CA inhibitors.

Molecular modelling was also performed to get insight into the different inhibitory activities of derivatives towards the CA I and II (off-target) and the tumour-associated isoforms CA IX and CA XII. Selenols moieties of allyl and propargyl were evaluated *in silico* as they were cleaved upon CA action. The compounds of series **a** and **b** showed a very good agreement between the geometrical parameters (bond length and bond angles) of the experimental solutions (X-ray data) and the *in silico* data (docking positions) of active **a** (allyl) and **b** (propargyl). However, these results do not explain the large different activity exhibited *in vitro*. Thus, it seems that the different hydrolysis rate of the compounds in the CA isozymes would be related to the difference in the activity between derivatives of the two studied series. The more compact conformation assumed by derivatives **1–19b** could allow an easier entry into the active sites, promoting faster hydrolysis and leading to a higher inhibitory activity compared to analogs of series **a**.
